# Can YouTube Be Considered a Reliable Learning Tool for Urology Trainees Seeking Information on Testicular Exploration?

**DOI:** 10.7759/cureus.47340

**Published:** 2023-10-19

**Authors:** Munir Al-Ghazawi, Mohammed Saad, Hamza Salameh, Samuel Amo-Afful, Seren Khasawneh, Rami Ghanem

**Affiliations:** 1 Urology, Barts Health National Health Service Trust, London, GBR; 2 Orthopedics, North Devon District Hospital, Barnstaple, GBR; 3 Urology, Dorset County Hospital, Dorchester, GBR; 4 Dentistry, Jordan University Hospital, Amman, JOR; 5 Urology, King Hussein Cancer Center, Amman, JOR

**Keywords:** surgical-education, youtube®, surgical skills, urology, testicular exploration

## Abstract

Introduction

YouTube, the world's largest video platform, hosts thousands of educational surgical videos that many trainees rely on to enhance their understanding and proficiency in various surgical procedures. Consequently, a crucial inquiry arises regarding the trustworthiness of these videos as a valuable resource for these trainees. In this article, we address this question by focusing on one of the most frequently performed surgical procedures in the field of urology and assessing the effectiveness of these videos as an educational tool for urology trainees (ST3+: Specialty Training Year 3 and above).

Methodology

We conducted a comprehensive search on YouTube for all videos related to 'Testicular Exploration'. After applying specific inclusion and exclusion criteria, we identified a total of nine eligible videos for analysis. These videos were assessed using the LAParoscopic Surgery Video Educational GuidelineS (LAP-VEGaS) scoring system, which categorized them into two distinct groups. The first group, known as the 'high-quality group', included videos that scored 11 points or higher according to the LAP-VEGaS scoring criteria. The second group, termed the 'low-quality group', consisted of videos that scored less than 11 points using the LAP-VEGaS scoring tool. Additionally, we collected data on various metrics, such as video view counts, duration, likes and dislikes counts, comments count, like ratio, view ratio, and power index, and performed a comparative analysis between the two aforementioned groups.

Results

Between April 2013 and September 2023, the selected videos exhibited an average total view count of 95,546±138,000. The videos had an average duration of 6.35±2.26 minutes. Furthermore, the mean values for both likes and dislikes were 461.55±581 and 2.89±2.86, respectively. In contrast, the mean like ratio, view ratio, and power index were 0.98±0.0112, 176,00±13,100, and 173.80±131, respectively. The mean LAP-VEGaS scores for videos related to testicular exploration were 9.94±2.05. It is noteworthy that the first group had a statistically higher number of dislikes; however, the view count, comments count, likes count, and view ratio were statistically lower in the same group.

Conclusion

Videos related to testicular exploration on YouTube exhibit notably low quality and do not serve as a valuable resource for urology trainees. Key factors such as video duration, total view count, and viewer interactions (including likes, dislikes, and comments) should not be relied upon as indicators of educational video quality. Consequently, it is advisable for urology trainees to refrain from using YouTube as a primary source for learning about testicular exploration. Instead, they should seek guidance and support from experienced senior colleagues, educational supervisors, or consultants to explore more reliable sources of information for this surgical procedure.

## Introduction

Electronic learning (E-learning) is increasingly recognized as an efficient fundamental teaching approach that warrants promotion across all educational levels [1]. Moreover, it is regarded as a cost-effective and broadly inclusive method for educating students when compared to conventional teaching methods [2]. While there may be notable differences among E-learning platforms, it remains just as effective as other training methods, if not more so [3]. Undoubtedly, training programs are integrating E-learning into their curricula, and online training and assessment are poised to become integral components of surgical training [4].

And with rapid technological advancements and emerging of video platforms that have countless surgical educational videos, which are an integral resource for surgical trainees looking to advance in their careers, a pressing need has developed to understand the usefulness and reliability of these videos and how they are considered as a good source of information. Likewise, within the field of Urology, there are a multitude of surgical procedures that urology trainees must familiarize themselves with in order to perform them safely and accurately.

To acquire the necessary understanding and skills for these procedures, trainees should rely on a consensus among senior consultant urologists regarding what constitutes a 'high-quality' source. Taking the testicular exploration as a prime example of that, which is considered one of the most common urological procedures that any urology trainee should be able to perform in order to provide the best and quickest care to patients in need, numerous videos pertaining to this procedure are available. Yet questions surrounding their reliability persist.

Utilizing YouTube videos for surgical education and training represents an inventive approach to instruct and acquaint learners with surgical procedures. The wide accessibility of YouTube offers an advantageous platform for educational content. Nevertheless, the absence of peer review in the current setup may result in deficient or erroneous content [5].

## Materials and methods

On September 24, 2023, at 14:00 UK local time, we searched for 'Testicular Exploration' on the YouTube search bar, and a total of 34 videos were identified and sorted based on the views count from the most to the lowest count. The upload date range for our videos was between April 19, 2013 and September 9, 2023. Out of 34 videos, nine included, five shorts (YouTube videos less than 60 seconds), one playlist, one song, and 18 other excluded videos. 

Our exclusion criteria applied and removed any video that was 'Short' (less than 60 seconds in duration), any videos related to animal scrotal exploration, any videos featuring planned surgery for known pathology, any videos showing only a small part of the procedure or positive findings only, any educational videos that did not show an actual procedure, and the playlist that technically had duplication of already included videos. 

All nine videos were collected, and each video was analyzed for video duration, views count, comments count, likes count, dislikes count, like ratio (sum of likes/sum of likes and dislikes), view ratio (view count/years since uploaded), power index ([view ratio * like ratio]/100), high-quality video resolution (1080p, also known as Full HD or FHD (full high definition and above)), skin-skin procedure, and LAParoscopic surgery Video Educational GuidelineS (LAP-VEGaS) score.

The LAP-VEGaS score was performed for each of the nine videos by two senior urologists known to have a great involvement in surgical education. Each score was achieved by answering nine questions that each granted either 0 points (not presented in video), 1 point (partially presented in video), and 2 points (completely presented). A minimum of 0 and a maximum of 18 for each video were expected. The scores were divided into two groups: the first group (high-quality videos) had scores equal to or more than 11 points and the second group (low-quality videos) had scores less than 11 points. We also calculated the video's views count, duration, both likes and dislikes count, comments count, like ratio, view ratio, and power index and compared the results across the abovementioned two groups using IBM SPSS Statistics for Windows, Version 28.0 (Released 2021; IBM Corp; Armonk, New York, United States). 

## Results

All nine videos obtained and analyzed as per the abovementioned methodology are shown in Table 1. Over a period extended between April 2013 and September 2023, the selected videos showed a mean of total views of 95,546±138,000. The total view count of all videos was 859,910, and the total duration was 57 minutes and 5 seconds.

**Table 1:** Characteristics of the videos included. LAP-VEGaS: LAParoscopic surgery Video Educational GuidelineS.

Data set	Result
Total views	859,910
Average of views count	95,546
Total duration of all videos	00:57:05
Average duration of all videos	00:06:21
Total likes	4154
Average likes count	461.55
Total dislikes	26
Average dislikes count	2.89
Total comments	637
Average comments count	70.78
Average like ratio	0.98
Average view ratio	17,600
Average power index	173.80
Average LAP-VEGaS score	9.94
Percentage of high-quality resolution videos	56%
Percentage of skin-skin videos	44%

The mean video duration was 6.35±2.26 minutes. The mean of both likes and dislikes was 461.55±581 and 2.89±2.86, respectively. On the other hand, the mean like ratio, view ratio, and power index were 0.98±0.0112, 17,600±13,100, and 173.80±131, respectively. The mean of the LAP-VEGaS scores for testicular exploration videos was 9.94±2.05. The percentage of videos with a resolution of 1080p FHD quality was 56%, and procedures that involved a skin-skin procedure were 44%.

Of the nine selected videos, three were high quality and six were low quality, making a ratio of 1:2 (one high-quality video for each two low-quality videos). The LAP-VEGaS score ranged from 5 for the lowest-scoring videos to 16 for the highest one. The dislike numbers were statistically higher for the first group; however, the views count, the comments count, the likes count, and the view ratio were statistically lower for the same group. The P-value was not significant for video duration, like ratio, and power index across both groups. All P-values are demonstrated in Table 2.

**Table 2:** High-quality videos versus low-quality videos based on the LAP-VEGaS score along with the statistical significance. LAP-VEGaS: LAParoscopic surgery Video Educational GuidelineS.

Variable	LAP-VEGaS score ≥11 (high-quality video)	LAP-VEGaS score <11 (low-quality video)	P-value (significance)
Mean views count	34,744	125,946	0.016 (significant)
Mean video duration (seconds)	346.33	397.66	0.976 (not significant)
Mean comments count	37.67	87.33	0.049 (significant)
Mean likes count	224.33	580.16	0.038 (significant)
Mean dislikes count	4.67	2.00	0.029 (significant)
Like ratio	0.98	0.99	0.983 (not significant)
View ratio	13,525	19637.50	0.023 (significant)
Power index	163.39	194.54	0.980 (not significant)

**Figure 1:**
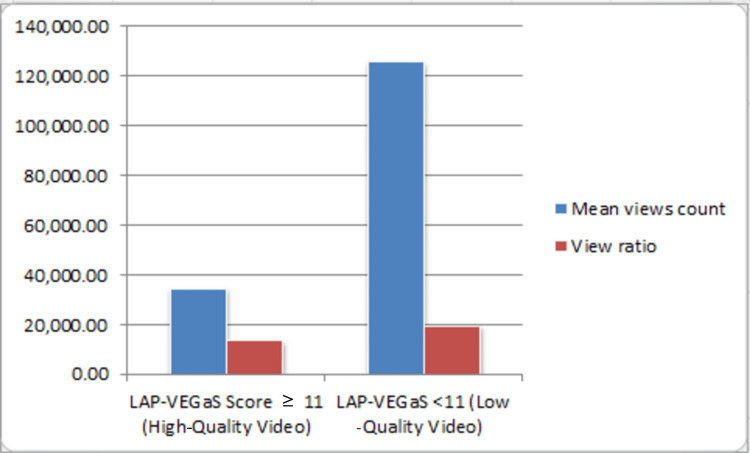
Mean views count and mean view ratio between high- and low-quality LAP-VEGaS scores. LAP-VEGaS: LAParoscopic surgery Video Educational GuidelineS.

Upon comparing the two groups by examining both the mean view counts and view ratio, it is clearly noted that they were statistically lower in the high-quality group, with P-values of 0.016 and 0.023, respectively. In Figure 1, the mean view count in the low-quality group was 125,946 compared to 34,744 views in the high-quality group. Alternatively, the view ratio was 19637.50 in the low-quality LAP-VEGaS group as opposed to 13,525 in the other group. 

**Figure 2:**
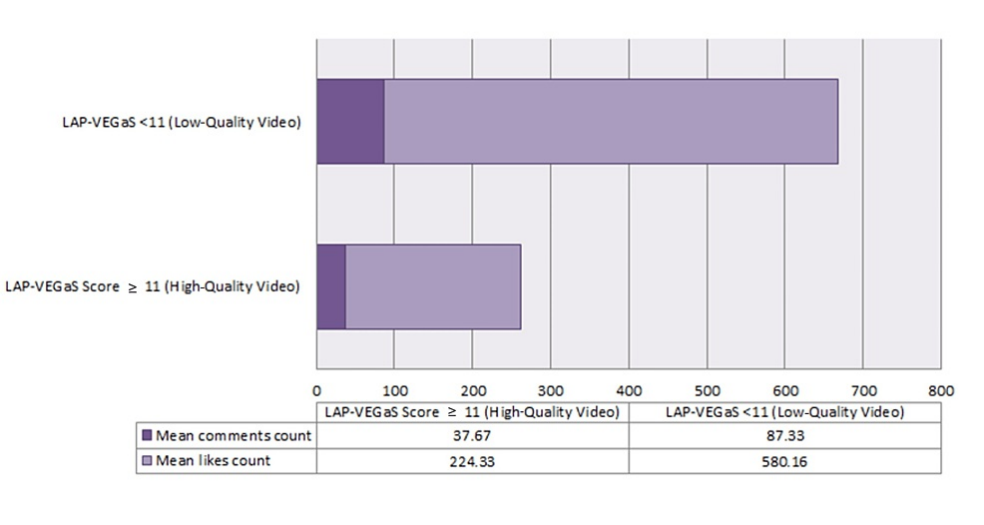
Interaction made on videos selected between two groups. Interactions: Included both likes and comments. LAP-VEGaS: LAParoscopic surgery Video Educational GuidelineS.

Figure 2 illustrates the difference between two groups based on the interaction made (likes and comments) on each of the nine videos, and again, it is obviously seen that both the mean likes count and mean comments count were statistically lower in the high-quality group, with P-values of 0.038 and 0.049, respectively. The mean total likes for the low-quality group was nearly double that of the high-quality group ( 580.16 vs. 224.33 ), and the mean comments count showed a quite similar ratio. 

## Discussion

We wish to elucidate the fact that the nomenclature of this assessment tool could potentially be misleading, given its explicit reference to laparoscopic surgery, whereas our current utilization involves the evaluation of open surgical procedures. It is noteworthy to mention that this tool was initially conceived and developed with a predominant focus on assessing laparoscopic surgeries and subsequently underwent modifications to accommodate its applicability to open surgical contexts. Furthermore, it is important to note that this assessment tool is specifically designed to assess the scientific content of uploaded videos rather than gauging their overall quality or resolution. The tool comprises nine questions meticulously tailored to address scientific aspects of the video, such as whether the procedure was executed correctly in a step-wise manner, if there was a clear description during the procedure, whether anatomical details were elucidated, if pathological aspects were identified, and whether preoperative and intraoperative findings were adequately mentioned, among other criteria.

The results of our study demonstrated a low quantity of testicular exploration videos on YouTube, and this is most probably due to uploading videos under other names like scrotal surgery, torsion surgery, and testicular surgery. The data showed a broad standard deviation across different parameters, indicating a significant variation in values among the videos that were assessed. For example, among the nine selected videos, three met the criteria for high quality, while the remaining six were considered low quality, resulting in a ratio of one high-quality video for every two low-quality ones. The LAP-VEGaS scores ranged from 5, the lowest, to 16, the highest, which again supports the wide range of quality and data gathered. Interestingly, the first group (high quality) had a statistically higher number of dislikes, but conversely, it had significantly lower counts in terms of views, comments, likes, and view ratios. And again, it is unexpected to have more dislikes for the highest quality videos, which makes the interactions on videos invalid factors to suggest whether this is of high or low quality. 

The utilization of the LAP-VEGaS score as a video assessment tool has the potential to elevate the overall caliber of published video content [6]. Many studies used the LAP-VEGaS score to assess YouTube videos as a useful source for surgical teaching. For example, Anand et al. showed that the videos found on YouTube for laparoscopic pyeloplasty in children were poor in quality as well [7]. Jones et al. tried to assess the availability of well-crafted YouTube videos explaining techniques for distal hypospadias repair, despite it being a common procedure, and once again, in this study, high-quality videos were rare [8]. On the other hand, Sunba et al. illustrated that the quality of YouTube videos serving as educational resources for thyroidectomy instructions varies. The majority of these thyroidectomy videos received a moderate quality rating based on the LAP-VEGaS score. Therefore, it is advisable to utilize YouTube thyroidectomy videos as a supplementary resource alongside traditional educational methods [9]. Taking the laparoscopic cholecystectomy as an example, Rouhi et al. demonstrated that although video walkthroughs offered by academic institutions exhibit superior LAP-VEGaS scores compared to other categories, they still fall short in meeting several crucial educational criteria for this procedure. This underscores the need for educators to make enhancements in these areas [10].

At this juncture, we may contemplate how to enhance the quality of these uploaded videos. Since there are no stringent criteria for uploading content to YouTube and not all uploaders are acquainted with the LAP-VEGaS scoring tool, we need to consider potential improvements. One suggestion is to promote education about this scoring system and ensure that uploaded videos explicitly mention their high-quality LAP-VEGaS score. Additionally, with technology constantly advancing, we propose the integration of such assessment tools into medical school curricula, for example, within biostatistics syllabi. This way, medical students and trainees can distinguish and disregard low-quality videos, allowing high-quality content to occupy a prominent position in search results. 

Another crucial consideration to be emphasized is the need for medical schools and training programs to allocate more resources to video-based learning, given its cost-effectiveness and remarkable efficiency along with face-to-face teaching in today's educational landscape. For instance, teaching a student how to perform a urinary catheter insertion can be accomplished effectively in a well-structured five-minute video, compared to the time-consuming process of explaining the procedure face-to-face, which undoubtedly takes longer than five minutes. Furthermore, students and trainees have the advantage of pausing and replaying sections they find challenging as many times as needed to improve their understanding, as opposed to merely observing the entire procedure once without addressing specific areas of weakness. It is essential to underscore that effective teaching should incorporate a blend of both virtual and in-person instruction.

Limitations 

Our study has a primary limitation stemming from the small sample size, primarily due to the scarcity of videos related to testicular exploration on the YouTube platform. This scarcity poses a challenge when it comes to establishing the true validity of specific results. Additionally, it's important to note the high popularity of the first two lower-quality videos, which contributed to a statistically lower P-value for the first group. These two videos focused on a critical step in testicular exploration, which is orchidectomy and fixation of the other testes. This particular aspect likely captured the interest of many urology trainees, resulting in their high view count and popularity.

Additionally, we want to emphasize that trainees often limit their online searches to a particular surgical procedure on YouTube. They spent a significant amount of time browsing through the search results instead of modifying the search query, such as using "scrotal surgery," which could be time-consuming and somewhat distracting. According to the LAP-VEGaS tool, the primary emphasis should be placed on a single, commonly searched topic by trainees. In our study, our primary focus was on testicular exploration surgery. Considering that we had over 30 videos, each lasting more than five minutes, trainees would dedicate a significant portion of their research time to these specific videos. And again, delving into scrotal surgery to locate instances of testicular exploration would consume considerable time and potentially introduce distractions.

Conversely, an additional constraint within our study could arise from the potentially inflated view counts of older, lower-quality videos in comparison to more recently uploaded ones. This discrepancy could potentially lead to erroneous findings in the correlation between video quality and view counts, as exemplified by the notably low P-value observed for high-quality videos.

Another limitation we encountered was the inability to establish a correlation between user interactions on the videos, including likes, dislikes, and comments, and the educational quality as measured by the LAP-VEGaS score. For instance, the video with the highest LAP-VEGaS score had more dislikes and fewer comments compared to some low-quality videos. Furthermore, the most viewed video had a LAP-VEGaS score lower than 11, indicating that these user interactions are not reliable indicators of content quality. It is worth noting that some videos may initially hide the dislike button to prevent negative impressions during the initial upload period.

All nine videos received a high score (+2) for the first question of the LAP-VEGaS tool, which assesses whether the title or procedure and pathology being treated are clearly documented. However, it is noteworthy that each of the nine selected videos received at least one zero score on certain questions, indicating that there was no single video that could be considered ideal in all aspects. Furthermore, it's important to mention that both consultants who assessed the LAP-VEGaS scores are highly experienced in the field of surgical education, which might have resulted in some conservative scoring. Nevertheless, the scores assigned to each video exhibited a consistent pattern, with a maximum difference of ±1 between the two scores, thereby ensuring that the overall LAP-VEGaS score was nearly accurate.

## Conclusions

The quality of YouTube videos related to testicular exploration is generally subpar, making them an ineffective resource for urology trainees. Metrics such as video duration, view count, and user interactions (including likes, dislikes, and comments) are not dependable indicators of the educational value of these videos. Consequently, it is advisable for urology trainees to refrain from relying on YouTube as a source for learning about testicular exploration. Instead, they should seek guidance and support from experienced senior consultants in order to acquire the necessary skills for this surgical procedure or explore alternative, more reliable sources of information.

## References

[1] Cooper D, Higgins S (2015). The effectiveness of online instructional videos in the acquisition and demonstration of cognitive, affective and psychomotor rehabilitation skills. Br J Educ Technol.

[2] George A, Blaauw D, Green-Thompson L (2019). Comparison of video demonstrations and bedside tutorials for teaching paediatric clinical skills to large groups of medical students in resource-constrained settings. Int J Educ Technol High Educ.

[3] Maertens H, Madani A, Landry T, Vermassen F, Van Herzeele I, Aggarwal R (2016). Systematic review of e-learning for surgical training. Br J Surg.

[4] Batirel HF, Assouad J, Etienne H, D'Journo XB (2021). Auditorium of the future: e learning platform. J Thorac Dis.

[5] Farag M, Bolton D, Lawrentschuk N (2020). Use of YouTube as a resource for surgical education-clarity or confusion. Eur Urol Focus.

[6] Celentano V, Smart N, Cahill RA (2021). Development and validation of a recommended checklist for assessment of surgical videos quality: the LAParoscopic surgery Video Educational GuidelineS (LAP-VEGaS) video assessment tool. Surg Endosc.

[7] Anand S, Jadhav B, Sandlas G (2021). Quality of YouTube Videos on laparoscopic pyeloplasty in children: an independent assessment by two pediatric surgeons. Cureus.

[8] Jones P, Rajasegaran A, Brassale S, Chen Y, Haslam R, Austin C, Seideman CA (2022). Assessment of the educational value of distal hypospadias repair videos on YouTube. Urology.

[9] Sunba S, Levin M, Wu V, Campisi P (2023). The educational value of thyroidectomy YouTube videos for surgical trainees. Am J Otolaryngol.

[10] Rouhi AD, Roberson JL, Kindall E (2023). What are trainees watching? Assessing the educational quality of online laparoscopic cholecystectomy training videos using the LAP-VEGaS guidelines. Surgery.

